# Zika Virus-Immune Plasmas from Symptomatic and Asymptomatic Individuals Enhance Zika Pathogenesis in Adult and Pregnant Mice

**DOI:** 10.1128/mBio.00758-19

**Published:** 2019-07-02

**Authors:** Byoung-Shik Shim, Young-Chan Kwon, Michael J. Ricciardi, Mars Stone, Yuka Otsuka, Fatma Berri, Jaclyn M. Kwal, Diogo M. Magnani, Cody B. Jackson, Audrey S. Richard, Philip Norris, Michael Busch, Christine L. Curry, Michael Farzan, David Watkins, Hyeryun Choe

**Affiliations:** a Department of Immunology and Microbiology, The Scripps Research Institute, Jupiter, Florida, USA; b Department of Pathology, University of Miami Leonard M. Miller School of Medicine, Miami, Florida, USA; c Viral Reference Laboratory and Repository Core, Blood Systems Research Institute, San Francisco, California, USA; d Department of Obstetrics and Gynecology, University of Miami Leonard M. Miller School of Medicine, Miami, Florida, USA; e Laboratory Medicine and Medicine, University of California, San Francisco, San Francisco, California, USA; Vanderbilt University Medical Center

**Keywords:** Zika virus, antibody-dependent enhancement, congenital disease, homotypic, microcephaly, pregnancy, vaccine, viral pathogenesis

## Abstract

Preexisting immunity against dengue virus or West Nile virus was previously reported to mediate antibody-dependent enhancement (ADE) of Zika virus (ZIKV) infection in a mouse model. We show here that ZIKV-immune plasma samples from both symptomatic and asymptomatic individuals mediated ZIKV ADE of infection *in vitro* and in mice. In a lethal infection model with a viral inoculum 10 times higher, both ADE and protection were observed, depending on the amount of infused immune plasma. In a vertical-transmission model, ZIKV-immune plasma infused to timed pregnant mice increased fetal demise and decreased the body weight of surviving fetuses. Depletion of IgG from an immune plasma abolished ADE of infection, and the presence of purified IgG alone mediated ADE of infection. Higher viral loads and proinflammatory cytokines were detected in mice treated with ZIKV-immune plasma samples compared to those receiving control plasma. Together, these data show that passive immunization with homotypic ZIKV antibodies, depending on the concentration, could either worsen or limit a subsequent ZIKV infection.

## Abstract (precis)

Antibody-dependent enhancement (ADE) of virus infection is common to many viruses and is problematic when plasma antibody levels decline to subneutralizing concentrations. ADE of infection is especially important among flaviviruses, many of which are the cause of global health problems. Recently, human plasma samples immune to heterologous flaviviruses were shown to promote Zika virus (ZIKV) infection. Here we showed in immunocompromised mouse models that homologous immune plasma samples protect mice from subsequent infection at high antibody concentrations but that they mediate ADE of infection and increase ZIKV pathogenesis in adult mice and fetal demise during pregnancy at low concentrations.

## INTRODUCTION

Zika virus (ZIKV) and dengue virus (DENV) are closely related in their sequences and structure. Infection with one DENV serotype typically results in short-term heterologous protection against other DENV serotypes. This protection subsides after only a few months, however, and a DENV infection with a different DENV serotype is 20 to 80 times more likely to result in severe disease conditions such as dengue hemorrhagic fever or dengue shock syndrome (1). This increase in disease severity is mediated by a mechanism described as antibody-dependent enhancement (ADE) of infection, and is best characterized for DENV, because there are four distinct and widely circulating DENV serotypes. ADE of infection occurs when antibody-coated viruses bind Fc gamma receptor (FcγR)-expressing cells and enter these cells through phagocytosis, subverting the process by which opsonized pathogens are normally cleared (2, 3). ADE of infection correlates with the decline in neutralizing antibodies. Subneutralizing concentrations of heterologous immune sera dramatically increase DENV infection of monocytes, macrophages, and dendritic cells—all major targets of flavivirus infection (4–6).

ADE of infection presents a significant hurdle to the development of safe and effective flavivirus vaccines. Experience with the tetravalent DENV-17D chimera vaccine Dengvaxia, developed by Sanofi-Pasteur and licensed for use in humans, highlights this challenge. Whereas this vaccine afforded 30% to 64% protection of the vaccinees with previous DENV exposure, the number of hospitalizations and severe dengue disease cases actually increased in the DENV-naive participants aged 2 to 9 years at the time of vaccination (7, 8). More recently, on the basis of follow-up studies performed up to 6 years postvaccination, the World Health Organization announced that this increased disease severity occurred regardless of the age of participants (9, 10). Thus, although vaccination boosted protective immunity in seropositive individuals, the vaccine rendered seronegative individuals more vulnerable to a dengue virus infection.

A number of recent studies have shown that anti-DENV plasma samples enhanced the pathogenicity of infections by ZIKV and that anti-ZIKV antibodies mediated DENV ADE of infection *in vitro* and *in vivo* (11–16, 68). In humans, ADE of infection is best observed in infants born to DENV-infected mothers (17–21). In addition to these examples of heterotypic ADE of infection, enhanced DENV infection mediated by antibodies or antisera directed against the same serotype (homotypic ADE of infection) was shown *in vitro*, albeit infrequently (6, 22, 23). Demonstration of homotypic ADE of infection in animals is scarce and controversial (24–26), but clinical cases from natural infections among humans, consistent with homotypic ADE of infection, are also found. For example, one DENV study showed that 4 of 29 reinfection cases were homotypic and exhibited much more severe symptoms than primary infection cases (27). In addition, babies born with maternal antibodies could be infected with DENV of the same serotype and developed dengue hemorrhagic fever/dengue shock syndrome (DHF/DSS), presumably because infection occurred in the presence of homotypic antibodies derived from the mother (19).

Although there are two lineages of ZIKV—African and Asian—they belong to a single serotype (28), and it is as yet unclear whether anti-ZIKV antibodies could mediate ZIKV ADE of infection. Here we show that ZIKV-immune plasma samples indeed enhanced the infection of ZIKV *in vitro* and *in vivo* and that plasma samples from both symptomatic and asymptomatic individuals mediated robust ZIKV ADE of infection. Higher viral loads and proinflammatory cytokines were detected in the blood and tissues of the animals infused with ZIKV-immune plasma samples. In a lethal-infection model, both ADE of infection and protection were observed with low and high concentrations of infused immune-plasma, respectively. Moreover, in a vertical-transmission model, infusion of immune plasma to timed pregnant dams resulted in increased fetal demise and smaller surviving fetuses. These data demonstrate that administration of ZIKV-immune plasma samples at high concentrations prevented infection but at low concentrations mediated homotypic ADE of infection and enhanced disease severity.

## RESULTS

### ZIKV-immune plasma samples induce ZIKV ADE of infection *in vitro*.

**FIG 1:**
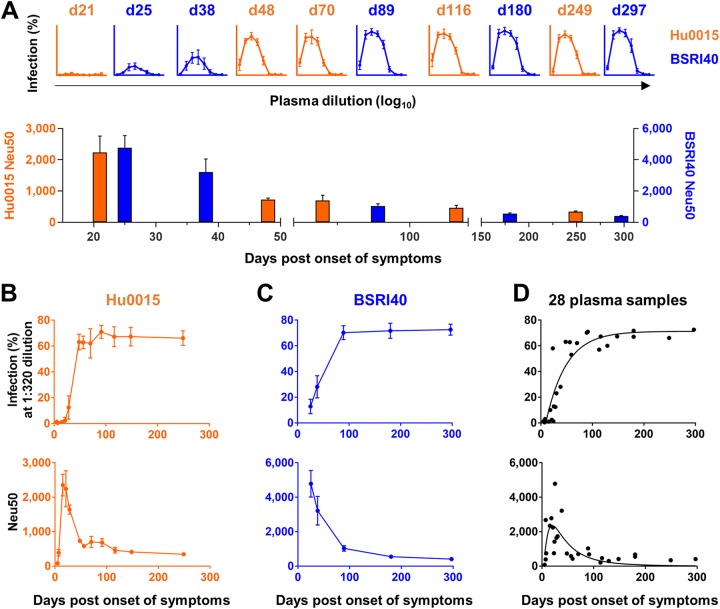
ZIKV-immune plasma samples induce ZIKV ADE of infection *in vitro*. (A) (Upper panel) ADE assays. K562 cells were infected at a multiplicity of infection (MOI) of 0.3 with ZIKV (PRVABC59) preincubated with serially diluted ZIKV-immune plasma samples derived from symptomatic individuals (Hu0015 and BSRI40). See Table 1 for information regarding ZIKV-immune plasma samples. Infection levels were assessed 72 h later by staining cells with the pan-flavivirus antibody 4G2. Under these conditions, ZIKV alone in the absence of added plasma yielded no detectable infection. The numbers above the data representing each ADE assay indicate days post-onset of symptoms (pos) at which plasma samples were collected. Additional ADE assay data are shown in Fig. S1. (Lower panel) Neutralization assays. Vero cells were infected at an MOI of 1 with ZIKV preincubated with serially diluted ZIKV-immune plasma samples. Infection levels were determined 24 h later by staining cells with the 4G2 antibody. The plasma dilution factors that resulted in 50% neutralization (Neu50) are shown. Additional neutralization assays are shown in Fig. S2. (B to D) Data from ADE (upper panel) and neutralization (lower panel) assays performed as described for panel A with ZIKV-immune plasma samples from Hu0015 (B) or BSRI40 (C) or with the 28 plasma samples derived from 10 symptomatic individuals (D) are shown. Averages ± standard deviations (SD) of results from three independent experiments are shown.

We have previously shown that ZIKV-immune plasma from a symptomatic ZIKV patient (Hu0015) enhanced ZIKV infection *in vitro* (29). Here we confirmed this observation using additional longitudinal plasma samples derived from two symptomatic ZIKV-positive individuals (Hu0015 and BSRI40; Table 1). No ADE of infection was observed for plasma samples collected at or before day 21 post-onset of symptoms (pos), and neutralizing capacity peaked between day 21 and d25 pos, gradually declining afterwards (Fig. 1A to C). To investigate if this observation was donor specific, we tested 11 additional ZIKV-immune plasma samples derived from 8 symptomatic individuals with no other flavivirus infection as determined by enzyme-linked immunosorbent assay (ELISA) or reverse transcription-PCR (RT-PCR) (Table 1). Again, no or little ADE of infection was detected with the plasma samples collected at or before day 18 pos, while ADE of infection was readily detected with those collected at or after day 23 pos (see Fig. S1 in the supplemental material). This ADE-mediating activity remained robust until day 297 pos. Like other plasma samples, neutralizing activity peaked early between day 15 and day 40 pos and declined thereafter (Fig. S2). The profiles of ADE of infection and neutralization of all 28 plasma samples from 10 individuals, determined as a function of days pos, are shown in Fig. 1D. These results show that ZIKV-immune plasma samples derived from symptomatic individuals, if collected 4 weeks or more after the onset of symptoms, induced ZIKV ADE of infection and that this ADE-mediating activity remained robust for at least 9 months thereafter.

**TABLE 1:** ZIKV-immune plasma samples included in this study^*a*^

Sample ID	Subject age (yrs)	Sex	Place of exposure	Collection time (days)^*b*^	Status	Diagnostic tests and results
Hu0002	28	M	Miami, FL	Not applicable	Healthy	Negative for ZIKV, DENV1–DENV4, and CHIKV by PCR and ELISA
Hu0007	53	M	Colombia	30	Symptomatic	Positive for ZIKV by PCR and ELISA, negative for DENV1–DENV4 by ELISA
Hu0015^*c*^	32	F	Miami (Wynwood), FL	5, 7, 15, 21, 28, 48, 56, 70, 91, 116, 148, 249	Symptomatic	Positive for ZIKV, negative for DENV1–DENV4 by PCR and ELISA
Hu0020	32	F	Miami (Miami Beach), FL	180	Symptomatic	Positive for ZIKV by ELISA and PRNT, negative for DENV1–DENV4 by ELISA
Hu0046	57	F	Cuba	7, 15, 24, 59	Symptomatic	Positive for ZIKV by ELISA, negative for DENV1–DENV4 and CHIKV by PCR
Hu0049	31	F	Miami, FL	247	Asymptomatic	Positive for ZIKV by ELISA and PRNT, negative for DENV1–DENV4 by ELISA
HuK021	36	F	Miami, FL	540	Asymptomatic	Positive for ZIKV by ELISA and PRNT, negative for DENV1–DENV4 by ELISA
HuK156	20	F^*d*^	Miami, FL	90	Asymptomatic	Positive for ZIKV by ELISA and PRNT, negative for DENV1–DENV4 by ELISA
UTMB3	26	F	Honduras	8	Symptomatic	Positive for ZIKV by ELISA
UTMB4	26	F	Caribbean Islands	18	Symptomatic	Positive for ZIKV by ELISA
UTMB7	15	F	Colombia	129	Symptomatic	Positive for ZIKV, negative for DENV1–DENV4 by PRNT
UTMB8	28	F	Colombia	113	Symptomatic	Positive for ZIKV, negative for DENV1–DENV4 by PRNT
UTMB134	60	F	Colombia	23	Symptomatic	Positive for ZIKV by PCR, negative for DENV1–DENV4 and CHIKV by PCR
BSRI39	43	F	Mexico	28, 41, 91, 180, 285	Asymptomatic	Positive for ZIKV, negative for DENV1–DENV4 by PCR and ELISA
BSRI40	26	M	NA	25, 38, 89, 180, 297	Symptomatic	Positive for ZIKV, negative for DENV1–DENV4 by PCR and ELISA
BSRI45	28	F	Puerto Rico	16, 87	Asymptomatic	Positive for ZIKV, negative for DENV1–DENV4 by PCR and ELISA

a ID, identifier; F, female; M, male; CHIKV, chikungunya virus; NA, not available.

b Plasma collection times indicate number of days after the onset of symptoms for symptomatic individuals and post-index donation, the number of days after the first collection day, for asymptomatic individuals.

c Plasma samples from this individual were previously studied for ZIKV-induced immunity (Ricciardi et al. [29]). Gray-shaded data represent samples derived from asymptomatic volunteers.

d Individual was pregnant.

10.1128/mBio.00758-19.1 FIG S1 *In vitro* ZIKV ADE assays with ZIKV-immune plasma samples. K562 cells were infected at an MOI of 0.3 with ZIKV strain PRVABC59 preincubated for 1 h with control plasma (Hu0002) or 17 ZIKV-immune plasma samples derived from 12 symptomatic individuals. Infection levels were assessed 72 h later by staining cells with the pan-flavivirus antibody 4G2. Under these conditions, the presence of ZIKV alone in the absence of added plasma yielded no detectable infection. Averages ± standard deviations (SD) of results from three independent experiments are shown. Download FIG S1, TIF file, 0.2 MB. Copyright © 2019 Shim et al. 2019 Shim et al. This content is distributed under the terms of the Creative Commons Attribution 4.0 International license.

10.1128/mBio.00758-19.2 FIG S2 *In vitro* ZIKV neutralization assays with ZIKV-immune plasma samples. Vero cells were infected at an MOI of 1 with ZIKV strain PRVABC59 preincubated for 1 h with control plasma (Hu0002) or 17 ZIKV-immune plasma samples derived from 12 symptomatic individuals. Infection levels were determined 24 h later by staining cells with the 4G2 antibody. Neu50, the plasma dilution factor that yield 50% neutralization, is indicated as a dashed line along with its value. Averages ± SD of results from three independent experiments are shown. Download FIG S2, TIF file, 0.2 MB. Copyright © 2019 Shim et al. 2019 Shim et al. This content is distributed under the terms of the Creative Commons Attribution 4.0 International license.

### ZIKV-immune plasma samples exacerbate ZIKV pathogenesis in mice.

**FIG 2:**
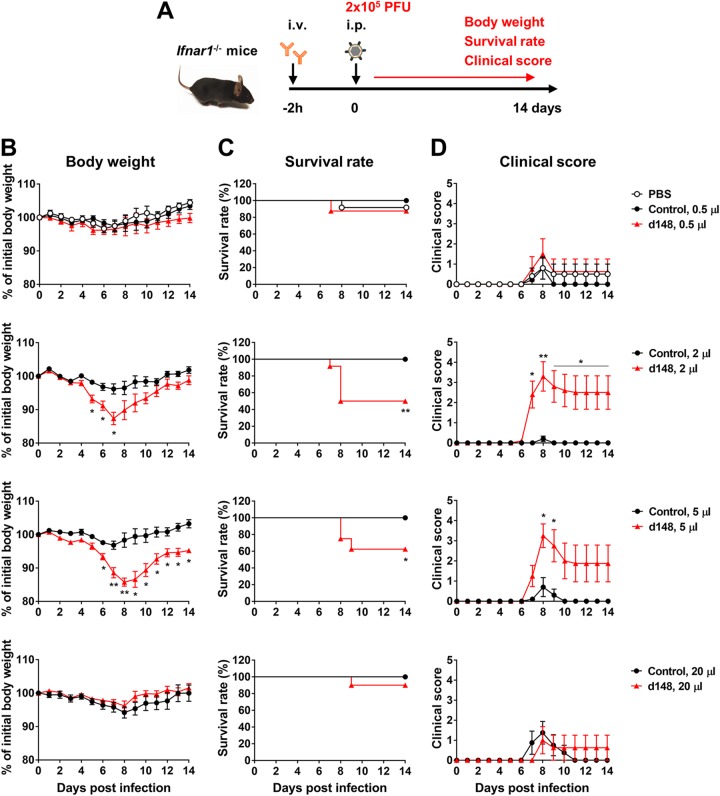
ZIKV-immune plasma samples exacerbate ZIKV pathogenesis in mice. (A) A schematic representation of an *in vivo* ADE assay. i.v., intravenous; i.p., intraperitoneal. (B to D) *Ifnar1*^−/−^ mice (*n* = 8 to 12 per group) were intravenously administered 0.5, 2, 5, or 20 μl of control plasma from a healthy individual (Hu0002) or ZIKV-immune plasma (Hu0015) collected at day 148 (d148) pos. PBS was also included in the 0.5-μl group. At 2 h later, mice were intraperitoneally infected with 2 × 10^5^ PFU of ZIKV (PRVABC59) and were monitored daily for body weight (B), survival (C), and clinical score (D) as described in Materials and Methods. Data shown in panels B to D are presented as means ± standard errors of the means (SEM). Significant differences between groups for body weight and clinical score were analyzed by multiple *t* tests using the Holm-Sidak method, and survival data were analyzed by log rank (Mantel-Cox) test. *, *P* < 0.05; **, *P* < 0.01.

**FIG 3:**
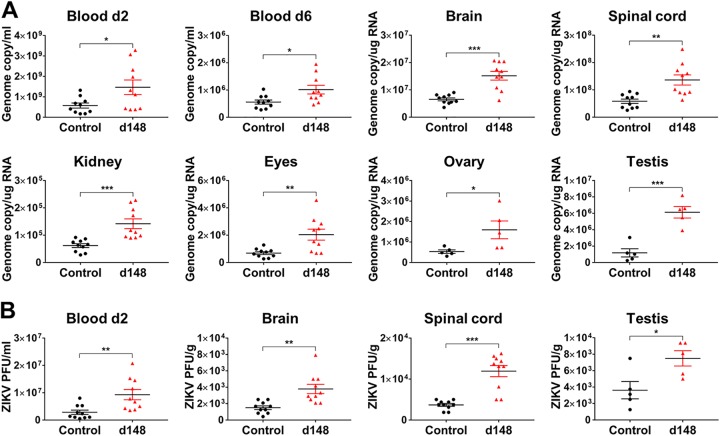
Viral loads are enhanced in blood and tissues of mice treated with ZIKV-immune plasma. (A) Mice (*n* = 10, or 5 each for ovary and testis) were intravenously administered 2 μl of control plasma (Hu0002) or day 148 (d148) pos plasma (Hu0015) and were infected as described for Fig. 2. Viral loads were determined by RT-qPCR in the blood at 2 or 6 days or tissues at 6 days postinfection (dpi). (B) Virus titers in the samples represented in panel A were determined by plaque assays. All data are presented as means ± SEM. Statistically significant differences between the groups were determined by an unpaired Student's *t* test. *, *P* < 0.05; **, *P* < 0.01; ***, *P* < 0.001.

To evaluate whether ZIKV-immune plasma samples can induce ZIKV ADE of infection *in vivo*, *Ifnar1^−/−^* C57BL/6 mice were intravenously infused with 0.5, 2, 5, or 20 μl of control plasma (Hu0002, healthy individual), or ZIKV-immune day 148 pos plasma (Hu0015) and were then infected with 2 × 10^5^ PFU of ZIKV strain PRVABC59 (Puerto Rico), as illustrated in Fig. 2A. Although mice treated with 2 or 5 μl plasma exhibited significant weight loss, those treated with a lower (0.5 μl) or much higher (20 μl) amount plasma displayed little weight loss (Fig. 2B), identifying a window in which the magnitude of ADE of infection is greater than that of neutralization. Mice receiving phosphate-buffered saline (PBS) or control plasma lost less than 5% of their initial body weight. While approximately 90% of the mice treated with 0.5 or 20 μl of immune plasma survived, only 50% or 60% of those receiving 2 or 5 μl immune plasma survived, respectively (Fig. 2C). No animal receiving control plasma died. Significantly higher clinical scores were observed with the mice infused with 2 or 5 μl of immune plasma than with those receiving 0.5 or 20 μl of the same plasma (Fig. 2D). To further investigate whether ZIKV ADE of infection in mice is associated with higher viral loads, we measured viral RNA levels in the blood and tissues of mice that received 2 μl of control or day 148 pos plasma samples. Viral RNA levels were 2-fold higher at 2 or 6 days postinfection (dpi) in the blood and were 2-fold to 5-fold higher at 6 dpi in the brain, spinal cord, kidney, eye, ovary, and testis of the mice receiving day 148 pos plasma than in those receiving control plasma (Fig. 3A). Comparable increases in viral loads were observed at 2 dpi in the blood and at 6 dpi in the brain, spinal cord, and testis as measured by plaque assays, but no infectious virus was detected in the kidney, eye, ovary, or blood (Fig. 3B). Although average viral loads increased only by 1.8-fold to 5.2-fold in the mice treated with immune plasma, the range of viral loads in these mice was much wider than the range in mice treated with control plasma. Thus, it is possible that the animals that died from severe pathogenesis were those who suffered from a higher degree of ADE of infection. Of note, the greatest increase in viral load was observed in the testis, an immune-privileged compartment important to sexual transmission of ZIKV.

**FIG 4:**
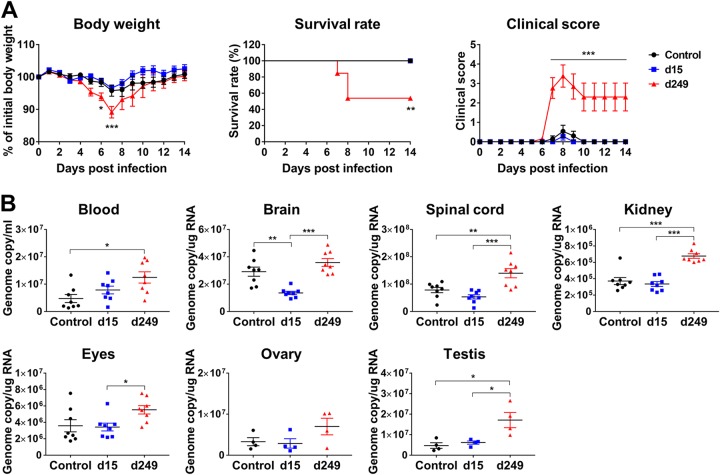
Immune plasma collected at an early time point after infection does not mediate ZIKV ADE of infection. (A) Experiments were performed similarly to those described for Fig. 2, except that 2-μl volumes of control plasma (Hu0002) or day 15 (d15) or d249 pos plasma samples (Hu0015) were assayed (*n* = 13 to 14). Data for body weight and clinical score were analyzed by two-way analysis of variance (ANOVA) using Tukey’s multiple-comparison test, and survival data were analyzed by log rank (Mantel-Cox) test. (B) Mice (*n* = 8 per group, 4 for testis) were intravenously administered 2 μl of control plasma (Hu0002) or day 15 or day 249 pos plasma samples (Hu0015) and were infected as described for panel A. Viral loads were determined in the blood and the indicated tissues at 6 dpi by RT-qPCR. All data are presented as means ± SEM. Statistical significance was determined by one-way ANOVA using Tukey’s multiple-comparison test. *, *P* < 0.05; **, *P* < 0.01; ***, *P* < 0.001. dpi, days postinfection.

We then used similar *in vivo* ADE assays to compare the plasma samples collected at early (day 15) or late (day 249) time points after the onset of symptoms. Consistent with the *in vitro* results, the mice receiving day 15 pos plasma did not exhibit any change in disease severity, while those treated with day 249 pos plasma exhibited more-severe ZIKV disease than the control mice (Fig. 4A). We also compared these plasma samples for their effect on tissue viral loads. As expected, while day 249 pos plasma resulted in increased viral loads at 6 dpi in the blood, spinal cord, kidney, and testis, day 15 pos plasma showed a slight increase in the blood only (Fig. 4B). Taken together, these results indicate that ZIKV-immune plasma samples can mediate ZIKV ADE of infection in mice and that the level of induction of ADE of infection is dependent on the amount of infused plasma and the collection time. These results also show that ZIKV ADE of infection in mice mediated by ZIKV-immune plasma samples is accompanied by increased viral loads in various tissues, especially in the testis.

### ZIKV-immune plasma samples from asymptomatic individuals also enhance ZIKV infection.

**FIG 5:**
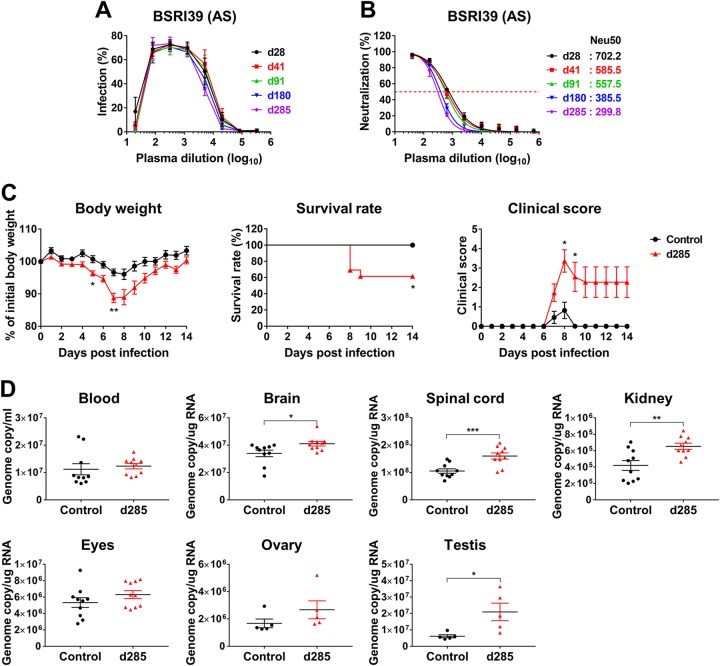
ZIKV-immune plasma samples from asymptomatic individuals also enhance ZIKV infection. (A) ADE assays were performed as described for Fig. 1A with ZIKV-immune plasma samples from an asymptomatic (AS) individual (BSRI39) collected on the indicated days post-index donation (pid), the number of days after the first collection of plasma. Additional ADE assays are shown in Fig. S3A. (B) Neutralization assays were performed as described for Fig. 1A. Neu50, the plasma dilution factor that yield 50% neutralization, is indicated as a dashed line alone, and its values are provided next to each sample identifier. Additional neutralization assays can be found in Fig. S3B. In panels A and B, averages ± SD of results from three independent experiments are shown. (C) Experiments were performed similarly to those described for Fig. 2B to D except that 2 μl of control (Hu0002, *n* = 11) and day 285 pid (BSRI39, *n* = 13) plasma samples were used. See Fig. S4 for additional data. Data for body weight and clinical score were analyzed by multiple *t* tests using the Holm-Sidak method, and survival data were analyzed by log rank (Mantel-Cox) test. (D) Mice (*n* = 10, or 5 each for ovary and testis) were infected with ZIKV as described for Fig. 2B, and viral loads were determined by RT-qPCR in the blood and tissues at 6 days postinfection. See Fig. S5 for the additional viral load data assessed by plaque assays. Data shown in panels C and D are presented as means ± SEM. Statistical significance was analyzed by an unpaired Student's *t* test. *, *P* < 0.05; **, *P* < 0.01; ***, *P* < 0.001.

Because an estimated 80% of humans infected with ZIKV are asymptomatic, we next asked whether plasma samples from asymptomatic individuals could also mediate ZIKV ADE of infection. Longitudinal plasma samples from an asymptomatic individual (BSRI39, Table 1) were assessed *in vitro* for their ADE-mediating and neutralizing capacities. As shown in Fig. 5A and B, the patterns of ADE of infection and neutralization mediated by these plasma samples were similar to those observed with the plasma samples derived from symptomatic patients. Similar results were obtained with additional plasma samples from four more asymptomatic individuals (Fig. S3A and B). We then further investigated whether these plasma samples were also able to aggravate ZIKV pathogenesis in mice (Fig. 5C). The mice infused with the plasma collected 285 days after the first donation (post-index donation [pid]) exhibited more severe ZIKV pathogenesis than those receiving control plasma. Specifically, we observed greater body weight loss, reduced survival, and increased clinical scores. ZIKV pathogenesis was also more pronounced in mice treated with day 540 pid plasma derived from another asymptomatic donor (Fig. S4). In addition, viral RNA levels were significantly higher at 6 dpi in the brain, spinal cord, kidney, and testis of the mice receiving day 285 pid plasma than in those of the mice treated with control plasma (Fig. 5D). Similar increases in viral loads were obtained by plaque assays in the spinal cord and testis (Fig. S5). The increase in viral RNA in the blood, eye, and ovary was not significant at 6 dpi, and no infectious virus was detected in those tissues. These results show that ZIKV-immune plasma samples derived from asymptomatic individuals also mediate ZIKV ADE of infection.

10.1128/mBio.00758-19.3 FIG S3 *In vitro* ADE and neutralization assays with ZIKV-immune plasma samples from asymptomatic individuals. ADE (A) and neutralization (B) assays were performed with five ZIKV-immune plasma samples from four asymptomatic individuals collected on the indicated days post-index donation (pid), as described for Fig. S1 and Fig. S2, respectively. The plasma dilution factors that yielded 50% neutralization (Neu50) in three independent experiments are shown as dashed lines, and data are presented as averages ± SD. Download FIG S3, TIF file, 0.2 MB. Copyright © 2019 Shim et al. 2019 Shim et al. This content is distributed under the terms of the Creative Commons Attribution 4.0 International license.

10.1128/mBio.00758-19.4 FIG S4 *In vivo* ZIKV ADE assays with ZIKV-immune plasma from an asymptomatic individual collected on day 540 post-index donation (pid). *Ifnar1*^−/−^ C57BL/6 mice (*n* = 14 to 16 per group) were intravenously administered 2 μl of control plasma (Hu0002) or day 540 pid plasma (HuK021). Mice were infected 2 h later via intraperitoneal injection with 2 × 10^5^ PFU of ZIKV strain PRVABC59 and were monitored daily for body weight (left panel), survival (middle panel), and clinical symptoms (right panel). Data are shown as means ± standard errors of the means (SEM). Significant differences between groups for body weight and clinical score were analyzed by multiple *t* tests using the Holm-Sidak method, and survival data were analyzed by log rank (Mantel-Cox) test. *, *P* < 0.05; ***, *P* < 0.001. Download FIG S4, TIF file, 0.1 MB. Copyright © 2019 Shim et al. 2019 Shim et al. This content is distributed under the terms of the Creative Commons Attribution 4.0 International license.

10.1128/mBio.00758-19.5 FIG S5 Tissue viral loads measured by plaque assays in mice treated with ZIKV-immune plasma samples from an asymptomatic individual. *Ifnar1*^−/−^ C57BL/6 mice (*n* = 10 per group, 5 for testis) were intravenously administered 2 μl of control plasma (Hu0002) or day 285 pid plasma (BSRI39) and infected as described for Fig. S4. Viral loads were determined in the indicated tissues at 6 days postinfection (dpi) by plaque assays. Data are presented as means ± SEM. Statistical significance of differences between the groups was determined by an unpaired Student’s *t* test. *, *P* < 0.05; ***, *P* < 0.001. Download FIG S5, TIF file, 0.1 MB. Copyright © 2019 Shim et al. 2019 Shim et al. This content is distributed under the terms of the Creative Commons Attribution 4.0 International license.

### In a lethal infection model, ZIKV-immune plasma samples mediate both ADE of infection and protection, depending on the infused amount.

**FIG 6:**
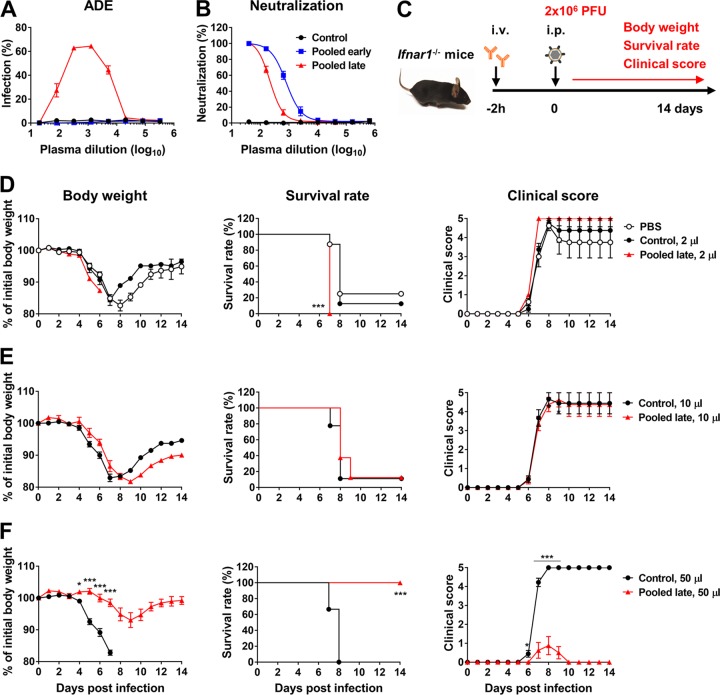
In a lethal infection model, ZIKV-immune plasma samples mediate both ADE of infection and protection depending on infused amount. (A) ADE assays were performed as described for Fig. 1A with ZIKV-immune plasma samples (HU0015) pooled for those collected on day 21 (d21) pos or earlier (pooled early) or for those collected on day 28 pos or later (pooled late). (B) Neutralization assays were performed as described for Fig. 1A. In panels A and B, averages ± SD of results from three independent experiments are shown. (C) A schematic representation of an *in vivo* ADE assay in a lethal infection model. i.v., intravenous; i.p., intraperitoneal. (D) *Ifnar1*^−/−^ mice (*n* = 8 per group) were intravenously administered 2 μl of PBS control (Hu0002) or pooled late plasma samples. At 2 h later, mice were intraperitoneally infected with 2 × 10^6^ PFU of ZIKV (PRVABC59) and were monitored daily for body weight, survival, and clinical score as described in Materials and Methods. (E and F) Infection experiments were performed similarly to those described for panel D except that mice were treated with 10 μl (E) or 50 μl (F) of control or pooled late plasma samples. Data shown in panels D to F are presented as means ± SEM. Significant differences between groups with respect to body weight and clinical score were analyzed by multiple *t* tests using the Holm-Sidak method, and survival data were analyzed by log rank (Mantel-Cox) test. *, *P* < 0.05; ***, *P* < 0.001.

All *in vivo* ADE assays up to this point were conducted with 2 × 10^5^ PFU of ZIKV per mouse. Under these conditions, ZIKV infection by itself caused a death rate of <10% (Fig. 2C, PBS group). After we verified that ADE of infection was readily detected with this ZIKV titer, we attempted to establish a lethal infection model, using a higher virus inoculum, in which both ADE of infection and protection would be able to be observed. In this experiment, immune plasma samples from symptomatic individuals were pooled into the following groups: those collected at or before day 21 pos (pooled early plasma) and those collected at or after day 28 pos (pooled late plasma). The patterns of the *in vitro* ADE of infection and neutralization assays determined using these pooled plasma samples (Fig. 6A and B) were similar to those determined using individual plasma samples (Fig. S1; see also Fig. S2). *Ifnar1^−/−^* mice were infused with 2 μl of PBS or 2, 10, or 50 μl of control or pooled late plasma and infected with 2 × 10^6^ PFU of ZIKV (PRVABC59) as outlined in the Fig. 6C legend. In this model, ZIKV infection alone resulted in 75% mortality by 8 dpi, but the remaining mice recovered and survived (Fig. 6D, PBS group). Whereas infusion of 2 μl pooled late plasma prior to infection increased mortality to 100% by 7 dpi with a worsened clinical score, infusion of 10 μl did not have a significant effect (Fig. 6D and E). On the other hand, infusion of 50 μl pooled late plasma protected mice and allowed 100% survival (Fig. 6F). These animals lost less weight, and their clinical score was much better than that of the animals treated with control plasma. These data underscore the point that antibodies in immune plasma samples can either mitigate or exacerbate disease severity, depending on the concentration.

### ZIKV-immune plasma samples increase fetal death and decrease fetal body weight in a vertical ZIKV transmission model.

**FIG 7:**
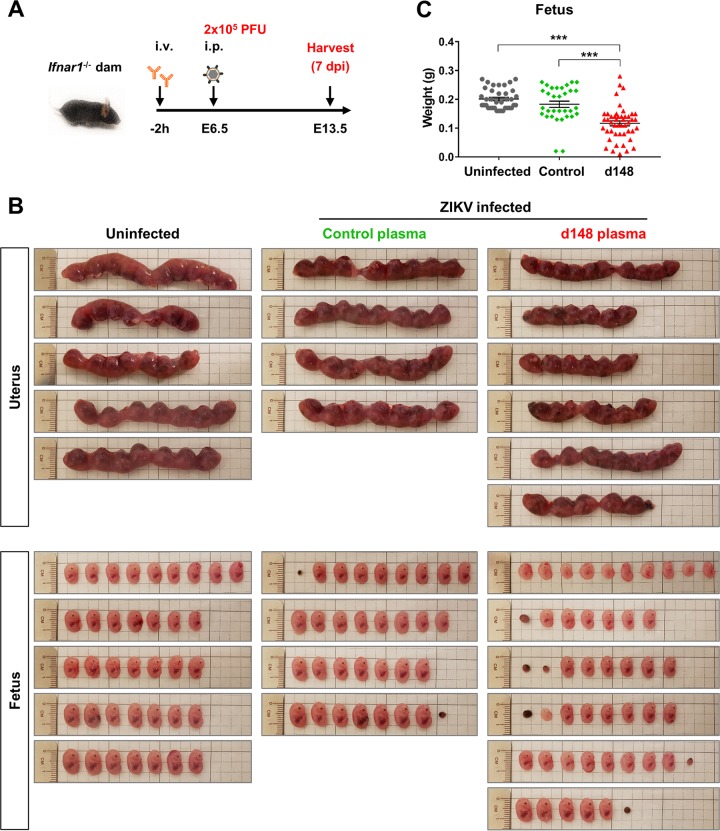
ZIKV-immune plasma samples mediated ADE of infection and decrease fetal body weight in wild-type and *Ifnar1^−/−^* vertical-transmission models. (A) A schematic representation of an ADE assay in a vertical ZIKV transmission model for ZIKV infection. i.v., intravenous; i.p., intraperitoneal. (B) On embryonic day 6.5 (E6.5), 7-week-old timed pregnant *Ifnar1*^−/−^ female mice (mated with *Ifnar1*^−/−^ males) were intravenously treated with 2 μl of control plasma (HU0002, *n* = 4) or day 148 pos plasma (HU0015, *n* = 6) and infected via an intraperitoneal injection 2 h later with 2 × 10^5^ PFU of ZIKV (PRVABC59). A group of timed pregnant dams (*n* = 5) were kept uninfected as a control. The uteri and fetuses from these dams were collected 7 days later on E13.5. (C) Body weights of the collected fetuses represented in panel B were compared. Statistical significance was determined by one-way ANOVA using Tukey’s multiple-comparison test. ***, *P* < 0.001.

Because the most severe consequence of a ZIKV infection is congenital malformation in newborns, we next investigated whether ZIKV-immune plasma samples would indeed worsen fetal defects in pregnant mice infected with ZIKV. Timed pregnant *Ifnar1^−/−^* female mice (mated with *Ifnar1^−/−^* males) were infused with 2 μl of control or day 148 pos immune plasma prior to infection with 2 × 10^5^ PFU of ZIKV (PRVABC59) on embryonic day 6.5 (E6.5) as illustrated in Fig. 7A. A group of timed pregnant mice were mock infected as a negative control. The uteri and fetuses were collected 7 days later, on E13.5. Whereas none of the 37 fetuses collected from 5 uninfected dams died, 2 of 32 fetuses from 4 dams were resorbed when dams were treated with control plasma prior to ZIKV infection (Fig. 7B). This result is consistent with human congenital infection and indicates that ZIKV infection by itself restricts normal fetal development. The rate for fetal demise was increased only moderately—7 of 48 from 6 dams—when dams were infused with immune plasma (Fig. 7B). However, a much greater difference was observed with fetus size, albeit with large variations among different litters (Fig. 7B and C). The average body weight of the fetuses from dams treated with immune plasma was significantly lower than that of the fetuses from dams treated with control plasma or left uninfected (Fig. 7C). These data show that maternal anti-ZIKV antibodies can mediate ADE of infection and exacerbate ZIKV-associated pathogenesis in the fetus.

### IgG-depleted ZIKV-immune plasma samples do not enhance ZIKV infection.

**FIG 8:**
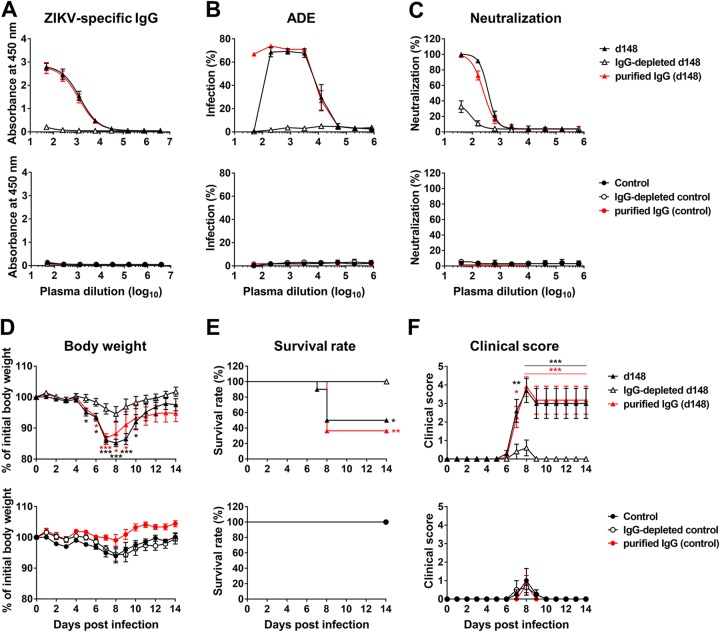
IgG-depleted immune plasma does not mediate ADE of infection. IgG-depleted ZIKV-immune or control plasma and purified IgG fractions were prepared from day 148 (d148) pos ZIKV-immune (Hu0015) or control (Hu0002) plasma, as described in Materials and Methods. The same volumes were used for IgG-depleted plasma samples, and an amount which contained a level of IgG equivalent to that in the original plasma was used for purified IgG. (A) ZIKV-specific IgG levels in the IgG-depleted plasma samples and purified IgG fractions were measured on a ZIKV-coated plate by ELISA. See Fig. S6 for ZIKV-specific IgG and IgM levels in the HU0015 longitudinal plasma samples. (B and C) ADE assays (B) and neutralization assays (C) were performed as described for Fig. 1A. In panels A to C, averages ± SD of results from three independent experiments are shown. (D to F) Experiments were performed similarly to those described for Fig. 2 except that 2 μl of control plasma, 2 μl of day 148 pos ZIKV immune plasma, equivalent volumes of IgG-depleted plasma samples, or equivalent amounts of purified IgG were used (*n* = 8 to 11 per group). Data are presented as means ± SEM. Significant differences between groups for body weight and clinical score were analyzed by two-way ANOVA using Tukey’s multiple-comparison test, and survival data were analyzed by log rank (Mantel-Cox) test. *, *P* < 0.05; **, *P* < 0.01; ***, *P* < 0.001.

To demonstrate that homotypic ADE of infection, like heterotypic ADE of infection, is mediated by IgG, we depleted day 148 pos ZIKV-immune plasma of IgG molecules using protein G-Sepharose. Protein G binds IgG molecules but not other classes of immunoglobulins. Depletion of IgG in the plasma was verified by ELISA for the absence of ZIKV-associated IgG (Fig. 8A). No ZIKV-specific IgG was detected in the control plasma. We observed that IgG depletion eliminated the neutralization and ADE-mediating capacities of the original day 148 pos plasma whereas IgG purified from the same plasma efficiently neutralized and mediated ADE of infection (Fig. 8B and C). IgG-depleted plasma was then compared to the original plasma and to the purified IgG in *in vivo* ADE assays. Whereas the mice treated with the IgG-depleted plasma lost less than 5% of the initial body weight, the mice receiving the original day 148 pos plasma or the IgG purified from this plasma suffered from significant weight loss (Fig. 8D, upper panel). None of the mice treated with control plasma or purified IgG from the control plasma lost weight (Fig. 8D, lower panel). Likewise, 64% or 50% of the mice injected with purified immune IgG or the original day 148 plasma, respectively, died, while all the mice treated otherwise survived (Fig. 8E). Similarly, data from the mice treated with the day 148 pos plasma or the IgG purified from this plasma exhibited clinical scores much higher than the scores seen with those treated with the IgG-depleted plasma (Fig. 8F). Low clinical scores were observed for the mice treated with control plasma.

10.1128/mBio.00758-19.6 FIG S6 ZIKV-specific IgG and IgM levels in the HU0015 longitudinal plasma samples and pooled early and late plasma samples. (A and B) (Left panels) Falcon 96-well plates were precoated with 100 μl of ZIKV (1 × 10^10^ genome copies/ml) diluted in Tris-buffered saline (TBS, pH 7.4) overnight at 4°C. After blocking with TBS containing 5% skim milk for 1 h at room temperature, a 100-μl volume of plasma or purified IgG samples serially diluted in blocking buffer containing 0.05% Tween 20 was added and incubated for 1 h at room temperature, followed by incubation with 1:3,000-diluted horseradish peroxidase-conjugated goat anti-human IgG (A) or goat anti-human IgM (B) for 1 h. The reaction was developed with tetramethylbenzidine and stopped by adding the same volume of 2 M phosphoric acid. (Right panels) Data represent the absorbance values determined as described for the left panel but at a 1:1,250 plasma dilution. Download FIG S6, TIF file, 0.2 MB. Copyright © 2019 Shim et al. 2019 Shim et al. This content is distributed under the terms of the Creative Commons Attribution 4.0 International license.

Note that although IgG depletion substantially reduced neutralizing activity from day 148 pos plasma, the neutralizing activity was not completely removed; approximately 10% to 30% of the original neutralizing activity still remained at low dilutions (Fig. 8C). As evidenced by the absence of ADE of infection mediated by this plasma (Fig. 8B), the source of neutralizing activity does not appear to have been IgG. In addition, whereas purified immune IgG mediated efficient ADE of infection at low dilutions, the original day 148 pos plasma at the same dilutions did not. A likely explanation for this outcome is that the original plasma contained neutralizing activity that was not associated with IgG. As moderate and low levels of virus-specific IgA and IgM, respectively, are known to circulate for at least 6 months following an infection (30), we measured ZIKV-specific IgM and IgG levels in the HU0015 longitudinal plasma samples as well as in pooled early and late plasma samples (Fig. S6). We observed that day 148 pos plasma contained IgM, albeit at a low level, explaining the presence of neutralizing activity in the IgG-depleted day 148 pos plasma (Fig. 8C). We further observed that, although ZIKV-specific IgG reached a maximum level at day 15 pos, IgM also reached a maximum level at day 15 pos (Fig. S6), providing an explanation for why the plasma samples collected at or before day 21 pos did not mediate ADE of infection (Fig. S1). These results demonstrate that, as with heterotypic ADE of infection, IgG is the plasma component that mediates homotypic ADE of infection and the presence of IgM can weaken ADE of infection.

## DISCUSSION

Although ADE of infection was first detected with two distinct DENV serotypes (31, 32), it can also promote infection when the serotypes are identical, at least *in vitro* (6, 22, 23). Considering the mechanism of ADE of infection, this is not surprising. When neutralizing antibodies are abundant, virus infection is inhibited. However, when the immune response is suboptimal or when it wanes, antibodies fail to neutralize viruses and instead promote virus infection via ADE of infection. This shift occurs when the number of antibodies bound to the virion is on average lower than a well-characterized threshold for neutralization (33). In addition, some antibodies, such as those targeting the flavivirus prM protein or certain epitopes of the E protein, are poorly neutralizing but efficiently mediate ADE of infection at all concentrations (5, 24, 34–36). These contributors to ADE of infection are also present when an infecting virus is of a serotype identical to that causing a previous infection, and there is no known mechanism that would establish a qualitative difference between homotypic and heterotypic ADE of infection. We show here that homotypic ZIKV ADE of infection is indeed possible and can substantially increase the severity of ZIKV disease in adult and pregnant mice. We observed homotypic ZIKV ADE of infection when mice were infused with 2 or 5 μl of plasma derived from ZIKV-infected individuals. We also showed in a lethal infection model with a higher virus inoculum that both ADE of infection and protection were observed with small and large amounts of infused immune plasma, respectively. These results confirm that observed ADE of infection *in vivo* was the outcome of the balance between neutralization and actual ADE of infection. They also suggest that care must be taken to ensure that subunit vaccines elicit a sufficiently high titer of neutralizing antibody in most individuals, especially given the wide variation in human immune responses.

In addition to the natural decline of immunity with time, most vaccines do not raise antibodies as potent as those observed in a natural infection, and the durability of protection provided by a vaccine varies across individuals (37). Although the case of Dengvaxia vaccine highlights the potential dangers associated with ADE of infection across DENV serotypes, there are currently insufficient epidemiological data to support the idea of ZIKV ADE of infection in humans except in a few reports from a limited number of studies (18). Furthermore, animal studies are controversial (11, 38–40, 68) owing to the need to find an appropriate range of antibody concentrations in which ADE of infection is more effective than neutralization. In particular, primate ADE studies have been further hampered by the necessity of using small cohorts. Epidemiological studies have shown that severe DENV diseases, likely caused by ADE of infection, occur in only 3% to 6% of individuals (41). Presumably, the proportion of immunocompetent rhesus macaques experiencing ADE of infection would be similarly low and thus would not be observed in underpowered studies.

Since the symptoms of ZIKV disease are generally mild, the major concern associated with ZIKV is the fetal defects caused by congenital infection, a phenomenon reproduced in mice (42–44) and nonhuman primates (45–49). Our current study demonstrated that ZIKV-immune plasma infused into pregnant mice increased the rate of fetal demise and decreased the body weight of the surviving fetuses upon ZIKV infection. It also showed that ZIKV ADE of infection significantly enhanced viral loads in the testis compared to control animals, suggesting that ADE of infection may promote sexual transmission, another major concern associated with ZIKV infection (50, 51). These data may provide insight into why some immune-privileged sites with limited antibody access, including the developing brain and testis, may harbor viruses long after they are undetectable in the bloodstream. Because immune-privileged sites tolerate the introduction of antigens without eliciting an inflammatory immune response, only a low level of antibodies is detected in those compartments. For example, the level of anti-HIV-1 antibodies in the testis was 100-fold lower than that detected in blood of 28 HIV-1-seropositive individuals. Similarly, levels of maternal antibodies detected in the fetal brain were substantially lower than those found in the fetal bloodstream (52). In both cases, the low level of antibodies may help enhance virus replication in FcγR-expressing cells such as neurons and astrocytes as well as in microglia in the brain (53) or testicular macrophages (54). Of note, the antibodies detected in the testis were only those of the IgG subclass (55), the only immunoglobulin subclass that mediates ADE of infection, as our study confirmed. Similarly, only members of the IgG subclass are transported from the mother to the fetus across the placenta, potentially contributing to the amplification of ZIKV in the placenta and fetus, which might help explain the preferential infection of the placenta and prolonged detection of ZIKV RNA in pregnant women and nonhuman primates (49, 56–58). Further studies on the potential role of FcγR-mediated ADE of infection in immune-privileged sites are warranted.

Apart from these privileged sites, the differences in viral loads in tissue between control mice and those whose infection was made more severe by ADE of infection were modest. These modest viral load differences contrast with the marked differences in morbidity and mortality rates between these groups. However, serum levels of proinflammatory cytokines were also upregulated in animals that exhibited ADE of infection (see Fig. S7 in the supplemental material). As other studies have shown (59), proinflammatory cytokines can exacerbate ADE-mediated ZIKV pathology.

10.1128/mBio.00758-19.7 FIG S7 ZIKV-immune plasma samples increase proinflammatory cytokines in ZIKV-infected mice. *Ifnar1*^−/−^ C57BL/6 mice (*n* = 9 to 10 per group) were administered the control (Hu0002) or day 148 pos ZIKV-immune (Hu0015) plasma and were infected with ZIKV as described for Fig. 2. The levels of the indicated proinflammatory cytokines and chemokines in the blood of infected animals were measured at 2 or 6 dpi by the use of a MCYTOMAG-70K mouse cytokine/chemokine multiplex assay kit. Dashed lines indicate detection limits. Data are presented as means ± SEM. Significance was analyzed by one-way ANOVA using Tukey’s multiple-comparison test. *, *P* < 0.05; **, *P* < 0.01; ***, *P* < 0.001. Download FIG S7, TIF file, 0.2 MB. Copyright © 2019 Shim et al. 2019 Shim et al. This content is distributed under the terms of the Creative Commons Attribution 4.0 International license.

Our studies here show homotypic ZIKV antibodies can mediate ZIKV ADE of infection and exacerbate ZIKV pathology, but several limitations of these studies should be considered in interpreting the results. First, immunocompromised *Ifnar^−/−^* mice, in which all experiments were conducted, imperfectly model the human case, and additional innate immune responses lacking in this murine model might have altered the outcomes observed here. Second, immune plasma samples were passively infused into mice in our studies, and therefore no additional adaptive immune responses were present to suppress an ADE-enhanced infection (60). Third, although we showed that small amounts of plasma samples mediated ADE of infection and that larger amounts lent protection, it is unclear how to extrapolate these data to human sera to predict human outcomes or how they compare to the antibody levels elicited by vaccination. Efforts have been made to understand the correlation between the neutralizing capacity of plasma antibodies in humans and the outcome of a subsequent infection (17, 18, 61, 62). Efforts have also been made to determine the role of T-cell responses in ZIKV infection. While many ZIKV vaccine strategies focus on eliciting neutralizing antibodies, T-cell vaccines and live-attenuated vaccines (63), which contain the nonstructural proteins of ZIKV, can elicit robust T-cell responses and can help limit viral replication despite ADE of infection (60, 64–66). Further studies focusing on the correlates of protection, including plasma-neutralizing capacity and T-cell responses following infection or vaccination, and on the outcome of a rechallenge, especially in pregnant animals, are clearly necessary.

## MATERIALS AND METHODS

### Ethics statement.

A total of 38 preexisting, anonymized ZIKV-immune human plasma samples derived from 15 volunteers were obtained from three different sources. Blood draws were carried out according to the guidelines of the Internal Review Board (IRB) of the respective institutes, after obtaining consent from the participants, and the use of these plasma samples in this study was reviewed by the IRB of The Scripps Research Institute (IRB 17-7012). Twenty-one plasma samples derived from 7 volunteers were obtained from the University of Miami Medical Center. These samples were designated “Hu#” or “HuK#,” in which “#” indicates a 3-digit or 4-digit number (Table 1). Five plasma samples derived from 5 volunteers were obtained from the World Reference Center for Emerging Viruses and Arboviruses (WRCEVA) at the University of Texas Medical Branch, Galveston, TX, and designated “UTMB#.” Twelve samples derived from 3 volunteers were obtained from the Blood Systems Research Institute (BSRI) in San Francisco, CA, and designated “BSRI#.”

All animal experiments in this study were carried out in accordance with the recommendations in the Guide for the Care and Use of Laboratory Animals of the National Institutes of Health and were performed in an animal biosafety level 3 facility. All protocols were approved by the Institutional Animal Care and Use Committee (protocol 16-029). Mice that lost 20% or more of their initial body weight were euthanized using CO_2_ asphyxiation followed by cervical dislocation, which is consistent with the recommendations of the Panel on Euthanasia of the American Veterinary Medical Association.

### Plasma samples.

Symptomatic donors were identified if they presented maculopapular rash, retro-orbital pain, joint pain, or general malaise. Asymptomatic individuals positive for ZIKV were identified from routine blood screening by PCR assays for ZIKV and DENV. The donors positive for virus infection were deferred from blood donation, and ZIKV-positive individuals were enrolled into a follow-up study. Blood samples from these individuals were confirmed for ZIKV-positive and DENV-negative results using ELISAs. For some samples, additional assays were performed such as the plaque reduction neutralization test (PRNT) for ZIKV or DENV to measure antibody titers against these viruses and ELISAs to identify chikungunya virus infection. Information on blood donors and their plasma samples is provided in Table 1.

### Cells and viruses.

Vero (African green monkey kidney cell line; ATCC CCL-81) cells were grown in high-glucose DMEM medium in the presence of 10% fetal bovine serum (FBS) and K562 (human myelogenous leukemia cell line; ATCC CCL-243) cells in RPMI medium containing 10% FBS. These cell lines were verified for the absence of mycoplasma and were cultured at 37°C with 5% CO_2_. ZIKV strain PRVABC59, isolated from a patient in Puerto Rico, was obtained from the WRCEVA and was propagated in Vero cells. Culture supernatants of the infected cells were harvested, filtered with 0.45-μm-pore-size filters, and frozen at −80°C. Virus titers were determined using plaque assays as described previously (67).

### *In vitro* ADE assays.

The ability of plasma samples to mediate ADE of infection was measured as previously described (34). Heat-inactivated plasma samples were serially diluted in RPMI medium containing 10% FBS, were preincubated for 1 h at 37°C with 1.5 × 10^3^ PFU of ZIKV strain PRVABC59 in a total volume of 50 μl, and added to 5 × 10^3^ K562 cells in 96-well plates using 50 μl RPMI medium containing 10% FBS. At 72 h later, cells were washed with PBS, fixed with 2% formaldehyde–PBS, permeabilized with 0.1% saponin–PBS–2% goat serum, and stained with the pan-flavivirus antibody 4G2, as previously described (67). Infection levels were read by the use of an Accuri C6 flow cytometer (BD Biosciences).

### *In vitro* neutralization assays.

The neutralizing potency of human plasma samples was measured using a flow cytometry-based neutralization assay (29). Briefly, heat-inactivated plasma samples were serially diluted and preincubated for 1 h at 37˚C with 5 × 10^4^ PFU ZIKV (PRVABC59) in a final volume of 50 μl. This virus and plasma mixture was added to 2.5 × 10^4^ Vero cells plated on a 96-well plate and was incubated for 1 h at 37˚C. Cells were washed, supplemented with fresh media, and grown for 24 h. Cells were then trypsinized with 0.25% trypsin, fixed, and permeabilized. Cells were stained with the antibody 4G2, and infection levels were analyzed by flow cytometry as previously described (67). The plasma dilution factor that yields 50% neutralization (Neu50) was calculated using a nonlinear regression analysis with GraphPad Prism 7.0 software (GraphPad Software, Inc.).

### ZIKV infection of *Ifnar1^−/−^* C57BL/6 mice.

*Ifnar1^−/−^* C57BL/6 mice were purchased from Jackson Laboratories and were bred and maintained under specific-pathogen-free conditions at the animal facility of The Scripps Research Institute, Jupiter, FL. For *in vivo* ZIKV ADE assays, 7-week-old *Ifnar1^−/−^* C57BL/6 mice were intravenously injected with the indicated volume of heat-inactivated ZIKV-immune or naive plasma samples diluted in 200 μl PBS. Similar numbers of male and female mice were used. These mice were infected 2 h later via intraperitoneal injection of 2 × 10^5^ PFU of ZIKV strain PRVABC59 (Puerto Rico) and were monitored daily for body weight, survival, and clinical symptoms. When a lethal infection model was used, mice were infected with 2 × 10^6^ PFU ZIKV. Clinical symptoms were scored from 0 to 5 as follows: 0 for no symptom; 1 for ruffled fur; 2 for partial paralysis of a limb; 3 for paralysis of one hind limb; 4 for paralysis of two hind limbs; 5 for paralysis of one or both front limbs in addition to both hind limbs or for mice that were moribund or euthanized. Mice that were moribund or lost 20% or more of their initial body weight were euthanized, using CO_2_ asphyxiation followed by cervical dislocation, and counted as deceased.

### ZIKV infection of timed pregnant dams.

*Ifnar1^−/−^* female mice (7 weeks old) were mated with *Ifnar1^−/−^* males, and mating was checked by the presence of vaginal plug. On E6.5, timed pregnant mice were intravenously infused with 2 μl of control or day 148 pos immune plasma, intraperitoneally infected 2 h later with 2 × 10^5^ PFU of ZIKV (PRVABC59), and monitored daily. Seven days later—on E13.5—mice were euthanized with CO_2_, and the uteri and fetuses were collected.

### Quantification of viral loads in blood and tissues of infected mice.

Blood was collected from the retro-orbital plexus of ZIKV-infected mice at 2 or 6 dpi, and serum was obtained by centrifuging coagulated blood at 3,000 rpm for 10 min. Tissues were harvested after mice were anesthetized with isoflurane and perfused with 20 ml of PBS to avoid blood contamination. Tissues were homogenized in 500 μl PBS in a FastPrep24 homogenizer (MP Biomedicals), and total RNA was extracted from 100 μl tissue homogenate or 50 μl serum using an RNeasy tissue kit (Qiagen). Extracted RNA was reverse transcribed to cDNA using a High-Capacity cDNA reverse transcription kit (Applied Biosystems), and viral RNA levels were quantified by quantitative PCR (qPCR) with ZIKV NS3-specific primers (forward, 5′-TTATGGACACCGAAGTGGAAG-3′; reverse, 5′-CACGCTTGGAACAAACCAAA-3′) and a probe (5′-TCAGGCTTTGATTGGGTGACGGAT-3′). Virus titers were also measured in sera or tissue homogenates by plaque assays as described previously (67).

### Depletion and purification of IgG from plasma.

A 400-μl volume of control or day 148 pos plasma was diluted 1:10 in PBS, mixed with 500 μl of washed protein G-Sepharose beads for 1 h at room temperature, and loaded into a column. The passed-through volume was reloaded into the column two more times, and the final pass-through volume was used as a IgG-depleted plasma. Protein G-Sepharose beads in the column were then washed twice with PBS, and bound IgG was eluted with 2 ml of 50 mM glycine (pH 2.5) into 2 ml of 1 M Tris-HCl (pH 7.4), washed, and concentrated using Centricon (Millipore) and used as purified IgG fractions. The IgG concentration in the original plasma samples was estimated by protein G-Sepharose immunoprecipitation followed by SDS-PAGE and Coomassie staining, and purified IgG was diluted in PBS at the same concentration as was used for the original plasma.

### Zika virus-specific IgG and IgM ELISA.

Falcon 96-well plates were precoated with 100 μl of ZIKV (1 × 10^10^ genome copies/ml) diluted in Tris-buffered saline (TBS;pH 7.4) overnight at 4°C. After blocking with TBS containing 5% skim milk was performed for 1 h at room temperature, 100 μl of plasma or purified IgG samples serially diluted in blocking buffer containing 0.05% Tween 20 was added and incubated for 1 h at room temperature, followed by 1:3,000-diluted horseradish peroxidase-conjugated goat anti-human IgG (Jackson Research Laboratories) or goat anti-human IgM (Southern Biotech) for 1 h. After washing with TBS containing 0.05% Tween 20, the reaction was developed by the use of the peroxidase substrate tetramethylbenzidine (Millipore) for 3 min and stopped by adding the same volume of 2 M phosphoric acid. The absorbance at 450 nm was read in SpectraMax Paradigm plate reader (Molecular Devices).

### Quantification of cytokines and chemokines in the blood of infected mice.

Cytokine and chemokine levels were determined in serum samples using customized MCYTOMAG-70K Milliplex MAP mouse cytokine/chemokine immunology multiplex assays (Millipore Sigma) for gamma interferon (IFN-γ), tumor necrosis factor alpha (TNF-α), interleukin-1β (IL-1β), IL-2, IL-6, IL-10, IL-12 [p40], IL-12 [p70], IP-10, monocyte chemoattractant protein-1 (MCP-1), RANTES, and MIP-1β. Serum samples were diluted 1:3 in serum matrix, and assays were performed according to the manufacturer’s instructions. Assays were read in Luminex 200 (Invitrogen) and were analyzed by the use of xPonent software (Millipore Sigma).

### Statistical analysis.

All data were analyzed with GraphPad Prism 7 software (GraphPad Software, Inc.). Values were considered statistically significant for *P* values below 0.05 and indicated as follows: *, *P* = <0.05; **, *P* = <0.01; ***, *P* = <0.001. Specific statistical analysis methods are described in the figure legends where results from those experiments are presented.
