# Supplementary figures and images for: Zika Virus-Immune Plasmas from Symptomatic and Asymptomatic Individuals Enhance Zika Pathogenesis in Adult and Pregnant Mice
Source: mBio. 2019 Jul 2;10(4):e00758-19. doi: 10.1128/mBio.00758-19 (PMC6606798; doi:10.1128/mBio.00758-19)

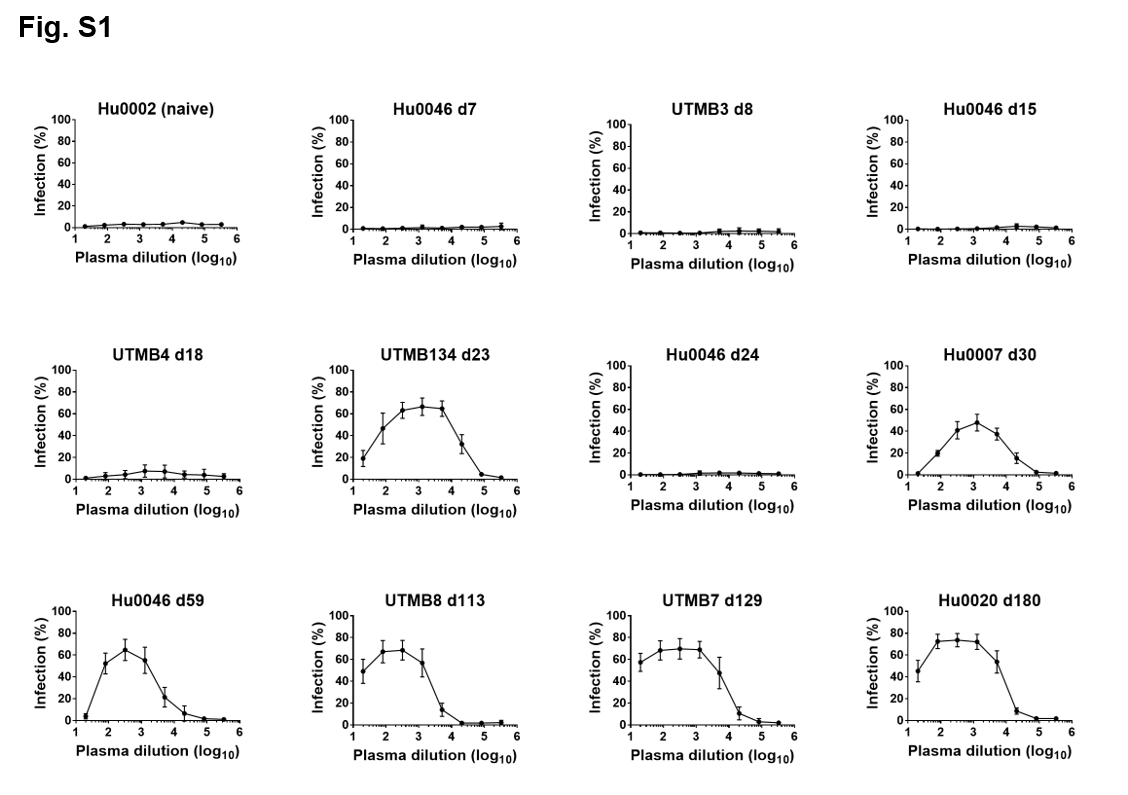

Supplement: FIG S1 [file mBio.00758-19-sf001.tif]

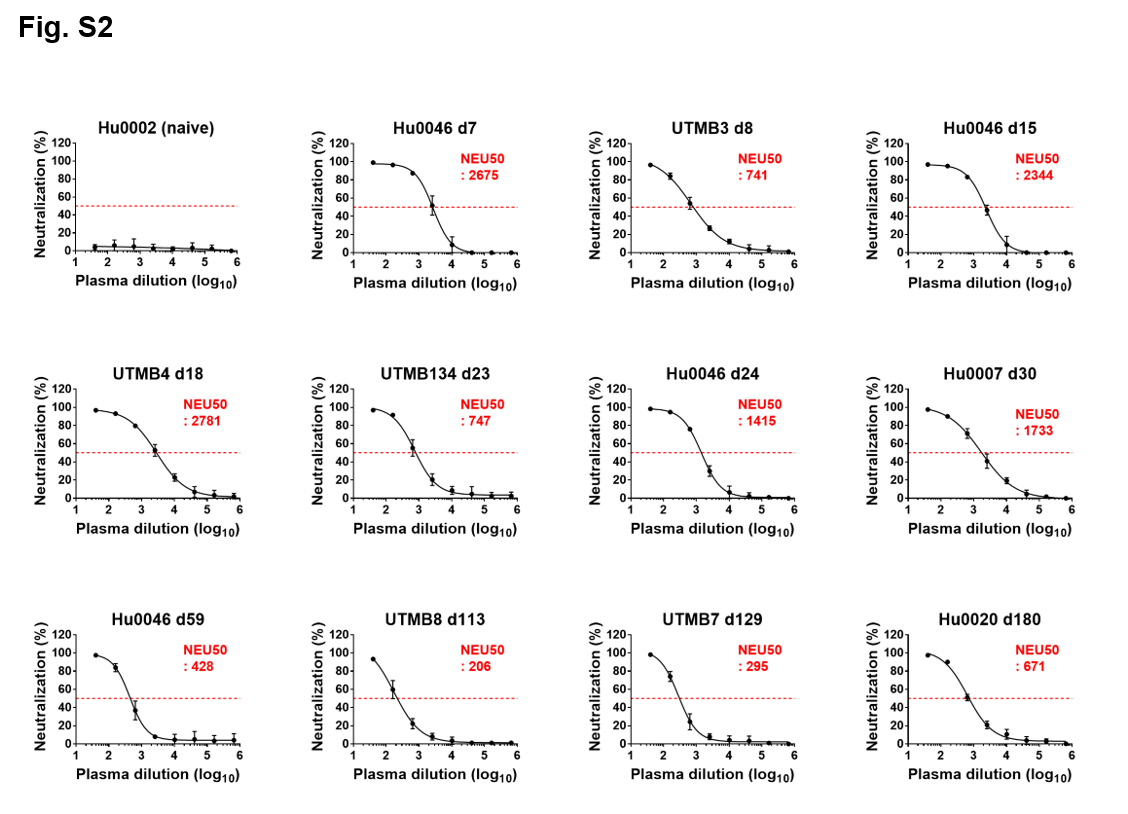

Supplement: FIG S2 [file mBio.00758-19-sf002.tif]

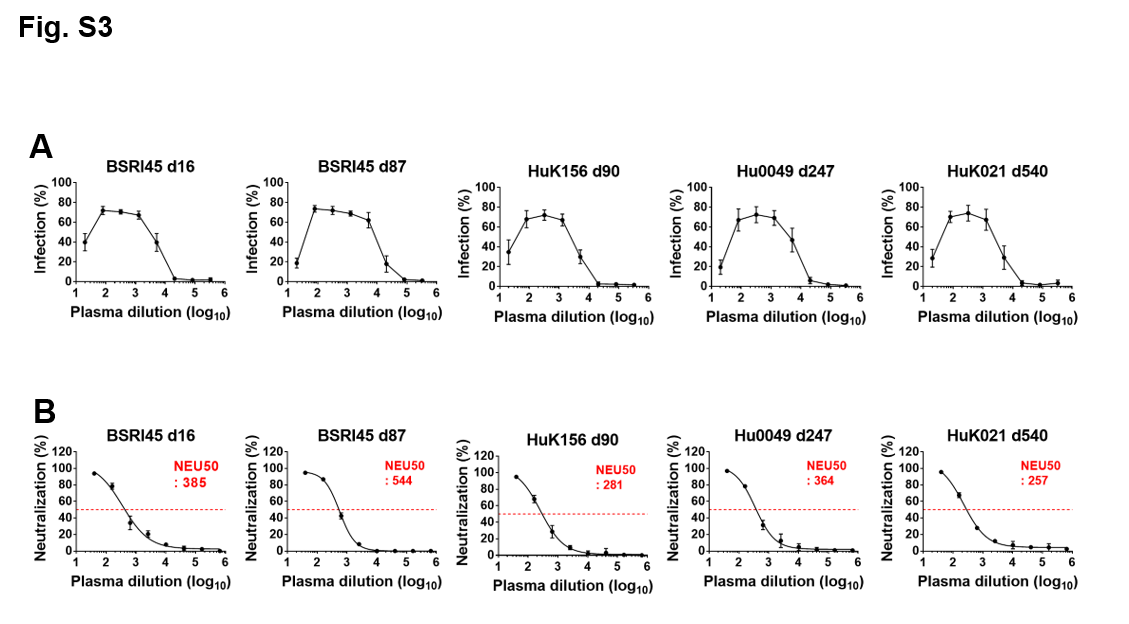

Supplement: FIG S3 [file mBio.00758-19-sf003.tif]

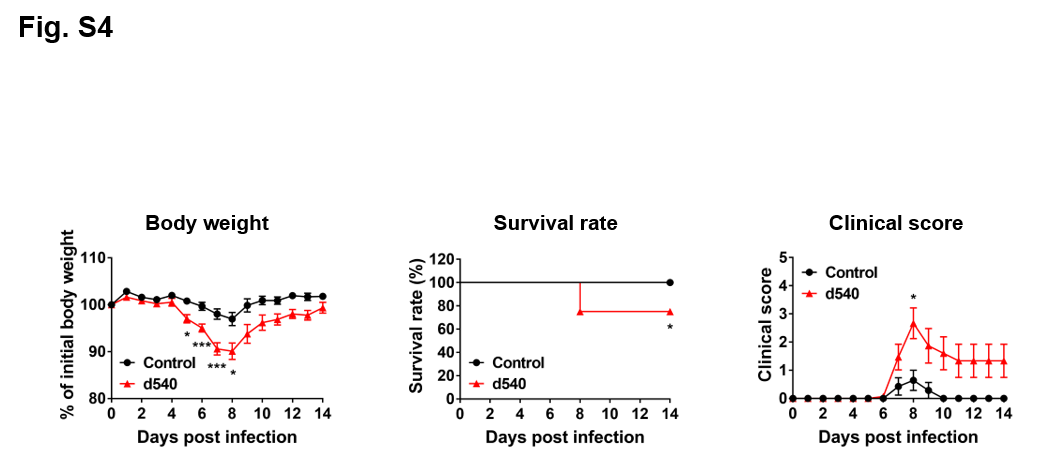

Supplement: FIG S4 [file mBio.00758-19-sf004.tif]

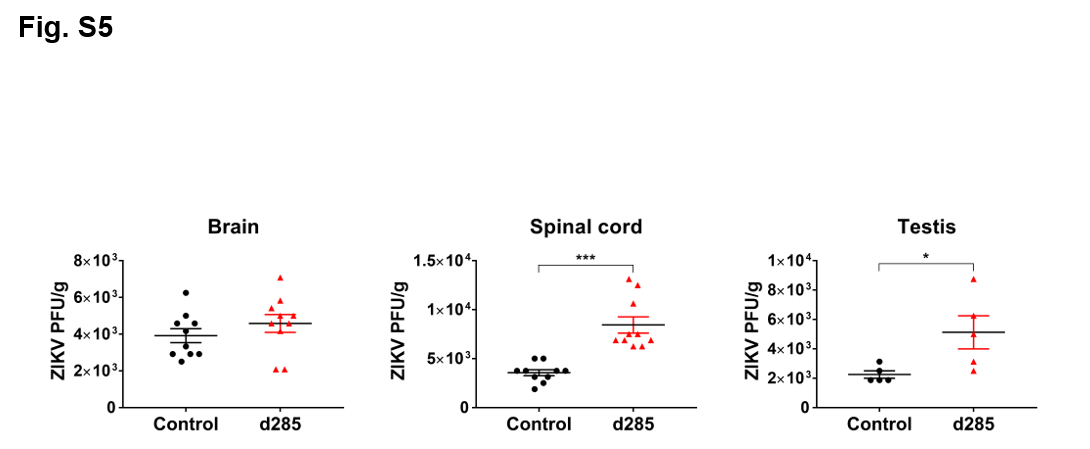

Supplement: FIG S5 [file mBio.00758-19-sf005.tif]

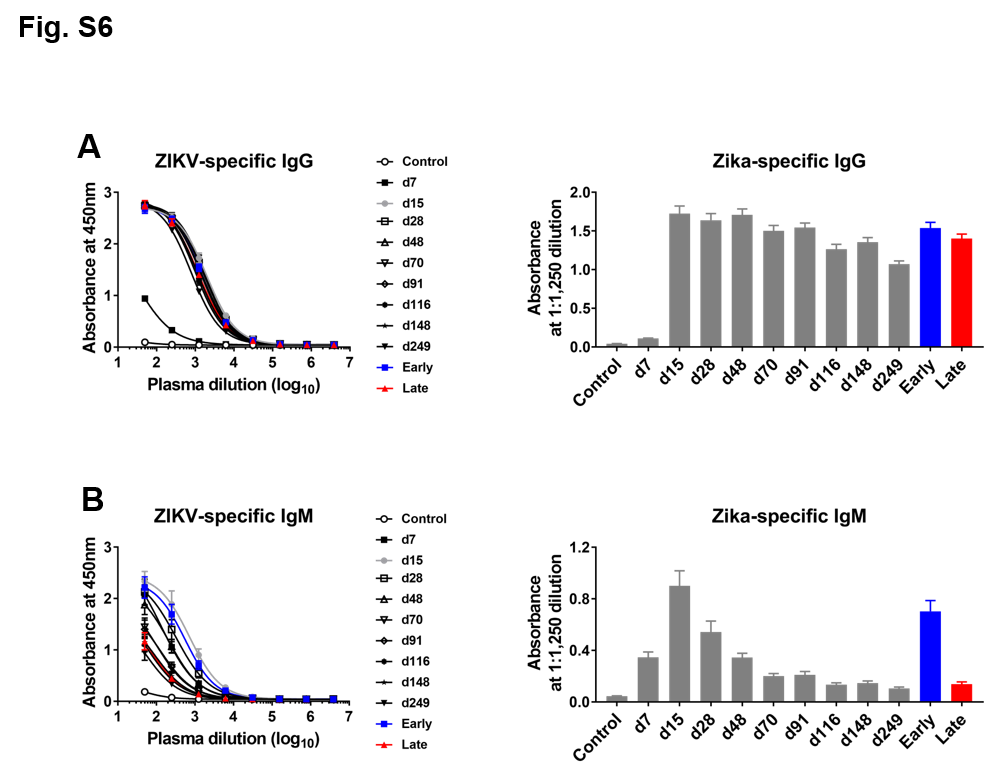

Supplement: FIG S6 [file mBio.00758-19-sf006.tif]

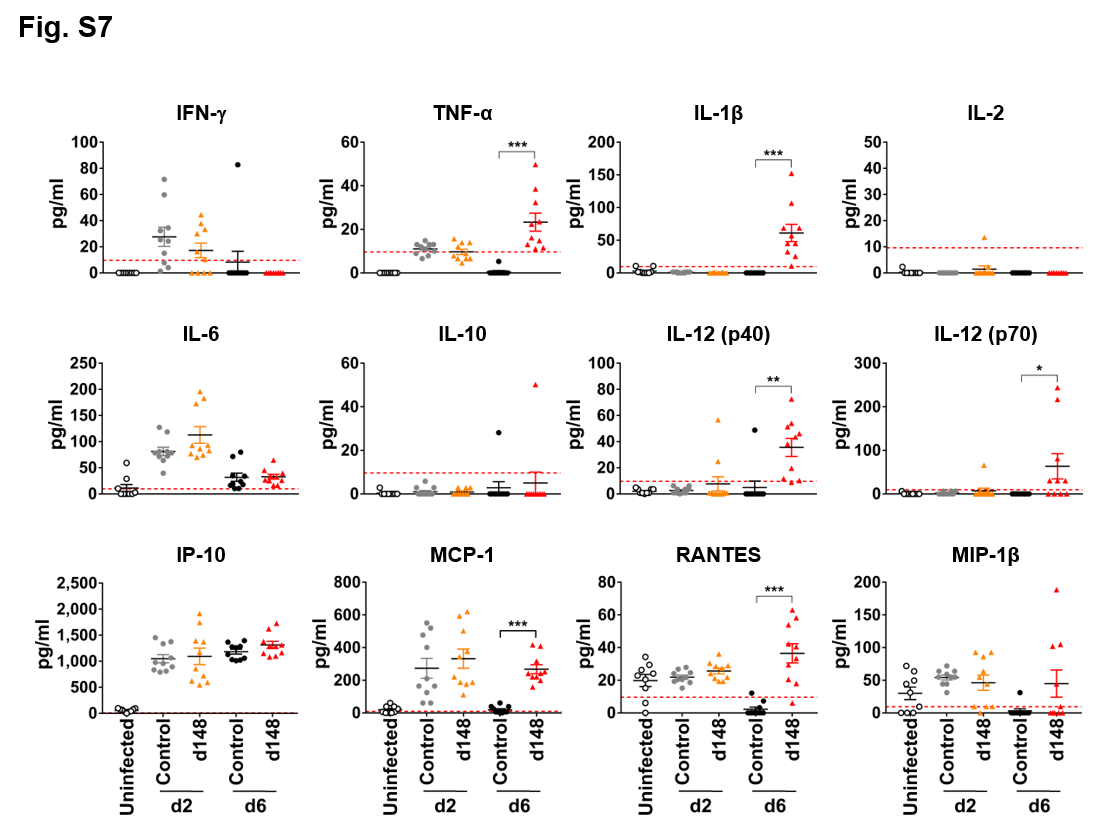

Supplement: FIG S7 [file mBio.00758-19-sf007.tif]
